# Effect of Silage Diet (Sweet Sorghum vs. Whole-Crop Corn) and Breed on Growth Performance, Carcass Traits, and Meat Quality of Lambs

**DOI:** 10.3390/ani11113120

**Published:** 2021-10-31

**Authors:** Pu Wu, Xiaoyue Fu, Hucheng Wang, Mingjie Hou, Zhanhuan Shang

**Affiliations:** 1 State Key Laboratory of Grassland Agro-Ecosystems, Engineering Research Center of Grassland Industry, Ministry of Education, College of Pastoral Agriculture Science and Technology, Lanzhou University, Lanzhou 730020, China; wup19@lzu.edu.cn (P.W.); xiaoyue0617@126.com (X.F.); houmj15@lzu.edu.cn (M.H.); 2 State Key Laboratory of Grassland Agro-Ecosystems, College of Life Sciences, Lanzhou University, Lanzhou 730000, China; shangzhh@lzu.edu.cn

**Keywords:** sweet sorghum, lambs, growth performance, meat quality, fatty acid

## Abstract

**Simple Summary:**

The increasing demand for better sensory characteristics, nutritional quality, and functional attributes of meat products that are beneficial to human health is stimulating the consumer market. Diet and breed directly affect ruminant carcass traits and meat quality. Therefore, this research aimed to evaluate the effects of silage diet and breed on growth performance, carcass traits, and meat quality of lambs. The lamb breed influenced fewer variables of growth performance and carcass characteristics compared to diet, and the lambs fed the sweet sorghum silage diet had higher nutritional quality meat than lambs fed the whole-crop corn silage diet.

**Abstract:**

Diet and breed directly affect ruminant carcass traits and meat quality. Therefore, this research aimed to evaluate the effect of silage diet and breed on growth performance, carcass traits, and meat quality of lambs. A total of 28, 3–4 months old female lambs consisting of 14 Dorper lambs (DP) and 14 Thin-tailed Han lambs (TH) were allocated in a 2 × 2 factorial design and offered two experimental diets (sweet sorghum silage: SS; whole-crop corn silage: WS) for 90 days. Lambs fed the WS diet had a higher growth performance (*p* < 0.01), intramuscular fat content (*p* < 0.05), and bright meat color (*p* < 0.01) than lambs fed the SS diet. The lambs fed the SS diet showed a higher polyunsaturated fatty acid (PUFA) content than the lambs fed the WS diet (*p* < 0.01); there was no significant difference in growth performance and carcass characteristics between DP and TH lambs (*p* > 0.05). The meat of the DP lambs showed lower values of initial pH, shear force, lightness (L*), redness (a*), and saturated fatty acid (SFA) content (*p* < 0.05). The lamb breed influenced fewer variables of growth performance and carcass characteristics compared to the diet. The lambs fed the SS diet had higher nutritional quality meat than lambs fed the WS diet.

## 1. Introduction

The increasing demand for better sensory characteristics, better nutritional quality, and functional attributes of meat products that are beneficial to human health is stimulating the consumer market. Meat production researchers are trying to meet these requirements by proposing more effective strategies and improving the sustainability and safety of the production system [1]. Moreover, it is confirmed that animals and their environment directly affect the carcass traits and meat quality [2]. Breed and diet are probably the most critical factors. Meat production in northwest China is mainly based on pure breeds of Dorper sheep (DP) and Thin-tailed Han sheep (TH) or their crosses. DP originated from South Africa and is characterized by hardiness, early maturity, and rapid growth [3], and the TH is a Chinese indigenous breed famous for its precociousness and prolificacy [4]; these are raised under an intensive production system. Whole-crop corn is the main ingredient of diets fed to lambs in the farms of northwestern China due to its optimum dry matter content at harvest and potential intake. However, there is an increasing cost of corn production due to some difficulties occurring in the area (e.g., groundwater shortages, mycotoxin contamination, plant attack by specific parasites) which are threatening agricultural sustainability, and large amounts of nitrogen fertilizers are needed to achieve satisfactory yields. In the last few years, many researchers have been trying to replace corn with other forages [5]. Sweet sorghum is an auspicious forage in the arid, semi-arid, and high salinity areas due to its high water-use efficiency, drought tolerance [6], high biomass yield (20–30 dry tons/ha), rapid growth rate, and phenolic compounds [7,8]. Some sweet sorghum varieties are characterized by moderate levels of phenolics, which has a positive effect on the efficiency of ruminant protein metabolism (e.g., a decrease in NH_3_–N losses) resulting in increased its performance, as well as improving ruminant milk and meat fatty acid composition [9,10,11]. In addition, silage can effectively preserve the nutrients of forage, and its use is not restricted by climate and season, so it has been widely used in the semi-arid and hilly area of the Loess Plateau in northwestern China. Experiments conducted on lactating cows have proven that the sweet sorghum silage (SS) can be a total replacement for whole-crop corn silage (WS) without undesirable effects on animal performance, but with positive effects on milk quality [12,13]. However, to the best of our knowledge, similar comparative information for SS and WS based diets are few, and no known research data are available on the effect of SS on meat quality of lambs, which is the subject of this study.

We hypothesized that the feeding potential of SS would be comparable with WS as sheep feed and it should have a positive effect on meat quality of lambs. To test the hypotheses, we examined growth performance, carcass traits, and meat quality of two dominant sheep breeds (DP vs. TH) fed with two different silage-type diets (SS vs. WS) in the semi-arid and hilly area of the Loess Plateau in northwestern China.

## 2. Materials and Methods

The experiment was conducted at Dingxi city of Gansu Province in China. All experimental procedures used in this study were conducted in accordance with the ethical guidelines approved by the Institutional Animal Care and Use Committee of Lanzhou University (10 May 2016).

### 2.1. Experimental Design and Animal Dietary Management

Twenty-eight female lambs from two breeds, DP (*n* = 14) and TH (*n* = 14), were 3–4 months old with an average live weight of 33.66 ± 0.37 kg and 25.57 ± 0.40 kg at the beginning of the experiment. Lambs of each breed were randomly assigned to two dietary treatments (*n* = 7) and adapted to their respective experimental diets for 10 days, followed by a 90 day feeding trial. Lambs were individually housed in covered pens with a concrete floor (length × width × height = 5.0 m × 2.5 m × 1.0 m) and they had free access to fresh water. During the experimental period, lambs were fed the following dietary treatments: SS) Sweet sorghum silage; WS) Whole-crop corn silage. According to the specific characteristics of each forage plant, sweet sorghum and whole-crop corn were collected at heading date and milk stage to obtain higher silage quality and economic benefits. Additionally, all the lambs received alfalfa pellets (collected at full-bloom stage) and concentrate which contained, on a dry matter basis, 41% corn meal, 10% wheat bran, 32% soybean meal, 5% corn gluten meal, 1% urea, 1% tone powder, 2% NaCl, and 8% commercial premix. Silage feed was provided ad libitum (10% minimum daily feed refusal allowed), the provided and refused amount of silage feed was recorded daily to adjust feed offered for 10% refusal. Concentrate and alfalfa pellets were provided twice daily, at 7:30 a.m. and 5:30 p.m., and provided based on the basis of 1% and 0.3% of their live weight. Both feed samples were taken weekly and frozen at −20 °C until analyzed. The day after feed administration, individual feed refusal was weighed to measure the average daily feed intake (ADFI). Lambs’ live weight was recorded at the beginning of the experiment and monthly on the same day and same time (7:00 a.m.) until the end of the trial in order to determined average daily gain (ADG). The ratio between average daily feed intake (ADFI) and average daily gain (ADG) was calculated as feed conversion ratio (F/G).

### 2.2. Feed Chemical Composition Analysis 

Feed samples were analyzed for dry matter (DM, method 934.01), crude protein (CP, method 968.06), and starch (Method 996.11) according to the official methods of analysis of the Association of Official Analytical Chemists [14]. The neutral detergent fiber (NDF), acid detergent fiber (ADF) and acid detergent lignin (ADL) were determined as described by Van Soest [15]. Total phenolics (TP) and total condensed tannins (CT) of SS and WS were measured using the Folin–Ciocalteu [16] and Osman [17] methods, respectively. Concentrate and alfalfa pellets were provided on the basis of 0.3% and 1% of live weight for all test animals, so the TP and CT content in these feed samples were not analyzed. Calculations were made to estimate diet metabolizable energy (ME, MJ/kg DM) [18] as follows:

ME = 0.046 + 0.820 × DE(1)

DE = 17.211 − 0.135 × NDF(2)

where ME is the diet metabolizable energy, DE is the diet digestible energy (MJ/kg), and NDF is the Neutral detergent fiber (%).

The nutrients of the diet are shown in Table 1.

### 2.3. Slaughter Procedure and Carcass Traits

At the end of the experimental period, all lambs were transported to an experimental abattoir where they were held overnight. Lambs were weighed after fasting for 24 h with free access to water to determine the slaughter weight and exsanguinated via jugular veins in compliance with veterinary police rules. After removing the skin, head, feet, and viscera, the carcasses containing kidney and pelvic fat were immediately weighed to obtain hot carcass weight (HCW). The dressing percentage was calculated by dividing the slaughter weight by carcass weight and expressed as a percentage. The carcasses were split along the spine, and a cross-section of the area between the 12th and 13th spinal ribs was drawn on sulfur paper. A planimeter (KP-21C; Koizumi Co. Ltd., Tsuchiura, Japan) was used to measure the loin muscle area. This procedure was repeated twice, and the average of the two measurements was recorded as the loin muscle area. A vernier caliper (0–1500 MM; Shengce Co. Ltd., Wengzhou, China) was used to measure the dorsal fat thickness (FT). Samples of longissimus dorsi (LD) were removed from the left half of the carcass. It was then divided into three subsamples. Two subsamples were immediately used to determine pH, color parameters, cooking loss, and shear force. One sample was vacuum-packed and stored at 4 °C for subsequent chemical composition (protein, fat, and ash), amino acid, and fatty acid analysis.

### 2.4. Physical-Chemical Analysis of Meat

The protein (method 984.13) and ash (method 942.05) contents of the meat were determined as described by the official methods of analysis of the Association of Official Analytical Chemists [14]. The intramuscular fat (IMF) content was determined by Soxhlet extraction [19]. Approximately 20 g of fresh LD was processed in a meat grinder (Philips HR2657, Eindhoven, Netherlands), lyophilized, and then pulverized. The powder (2 g) was extracted with petroleum ether and analyzed for lipids using the Soxtec 2055 fat extraction system (Foss Tecator AB, Foss, Sweden). The pH was measured at 45 min (pH_45 min_) and 24 h post mortem (pH_24 h_) with a penetrating electrode connected to a portable pH meter (Russell CD700; Russell pH Limited, München, Germany). Three readings were taken from each muscle, the pH values were averaged and the means were subjected to appropriate statistical analysis. Before pH measurement, the pH meter and electrode were standardized commercial pH buffers with 4.0 and 9.0 at room temperature (23 °C). For meat color parameters, a colorimeter (Minolta CR-300, Minolta Camera Co. Ltd., Osaka, Japan) was used to measure lightness (L*), redness(a*), and yellowness (b*) directly on the LD muscle surface in three different places 24 h post-slaughter; the measured area diameter was 8 mm and measurements were taken three times for each sample. Drip loss was measured by the LD muscle, which weights 20 g and was hung for 24 h at 4 °C in a closed plastic bag. The weight of each sample was recorded before and after the hanging treatment, and weight loss results were expressed as a percentage of the original weight. For cooking loss and shear force determination, the initial weight of the same LD muscle sample was weighed (Wi), stored in tin foil bags, then immersed in a water bath and heated for 30 min until the internal temperature reached 75 °C, which was monitored with a thermocouple (PT100; Subate Technology Co. Ltd., Shenzhen, China). Then the bags were placed under running tap water to cool for 30 min and blotted dry with paper towels. The cooked meat was finally weighed (Wf) and cooking loss was calculated as (Wi − Wf)/Wi × 100%. After cooling at room temperature (23 °C), a cork borer was used to remove six cores of 1.27 cm diameter and 2 cm long from each LD muscle to the muscle fibers. A material testing machine (Lloyd Instrument LRX plus, AMETEK Co. Ltd., Kitchener, ON, Canada) fitted with a shear blade travelling at 225 mm/min was used to cut each core once across the middle perpendicular to the fiber direction. The shear force was expressed in Newton (N) and values were averaged to obtain a mean value for each muscle.

### 2.5. Meat Hydrolysate Amino Acids Profiles

The LD samples were freeze-dried with a lyophilizer (Scientz-10ND; Ningbo Scientz Biotechnology Co. Ltd., Ningbo, China). The amino acid levels were determined according the procedure described by Tian et al [20]. The 30 mg LD sample was hydrolyzed in 10 mL HCl (6 mol/L) hydrolysis tube in a constant temperature drying oven at 110 °C for 22 h to 24 h. Hydrolyzates were diluted using 0.02 mol/L HCl in 50 mL volumetric flasks. After mixing, 1 mL solution was evaporated to dryness in a water bath at 65 °C. After dissolving in 2 mL HCl (0.02 mol/L), amino acids were fractionated on a protein hydrolysate analysis column (HISCO-855-4506; Hitachi, Tokyo, Japan) at an outflow rate of 0.40 mL/min. The individual amino acids were identified by comparison with standards (013–08391; Wako, Tokyo, Japan).

### 2.6. Intramuscular Fatty Acid Profiles

Lipids in the LD were directly methylated following the method of O’Fallon et al [21]. The LD samples were freeze-dried with a lyophilizer (Scientz-10ND, Ningbo Scientz Biotechnology Co. Ltd., Ningbo, China) and ground in a grinder (Philips HR2657; Eindhoven, The Netherland). Then, 0.7 mL of KOH (10 mol/L) and 6.3 mL of methanol were added and placed in a 55 °C water bath for 1.5 h, and shaken for 5 s every 20 min to make the sample hydrolyze normally. After cooling at room temperature, 0.58 mL H_2_SO_4_ (5 mol/L) was added and the tube turned upside down, then placed in a 55 °C water bath for 1.5 h. After FAME was formed, 2 mL n-hexane was added. The fatty acid profile was analyzed using a gas chromatograph (Thermo Fisher Scientific; Boston, Massachusetts, USA), which was equipped with a silicate glass capillary column 70% Cyanopropyl Polysilphenylenesiloxane BPX 70 of SGE Analytical Science (length = 50 m, internal diameter = 0.22 mm, film thickness = 0.25 m). The temperature procedure was as follows: 135 °C for 7 min, then increased by 4 °C to 210 °C per minute and held for 2 min. The temperature of the injector and detector were set up at 245 °C and 280 °C, respectively. Using helium as a carrier, the flow rate was 1.2 mL/min. Fatty acids were identified using standard fatty acid mixtures (Restek Corporation, Bellefonte, PA, USA) and expressed as a percentage of total methylated fatty acids.

### 2.7. Statistical Analysis

Referring to the procedures described by Horcada et al. [22], nutrient intake, growth performance, carcass traits, and meat quality were subjected to an analysis of variance (ANOVA). The effects of diet (SS and WS), breed (DP and TH), and their interactions (2 × 2) were evaluated using the GLM procedure of SPSS 21.0 (SPSS, Chicago, IL, USA), in order to avoid the effect of the initial weight within breed on nutrient intake, growth performance, and carcass characteristics, using initial weight as a covariate. The statistical model is as follows:

Y_ijk_ = u + B_i_ + PS_j_ + B_i_ × PS_j_ + e_ijk_ (3)

where Y_ijk_ = nutrient intake, growth performance, carcass traits, and meat quality; u = least squares mean value; B_i_ = fixed effect of diet (j = 1: SS; j = 2: WS); PSj = fixed effect of breed (i = 1: DP; i = 2: TH); B_i_ × PS_j_ = interaction between the diet effect and breed effect; eijk = random residual. Individual lamb was considered as the experimental unit. Residual mean squares were used as error term. Data were shown as the mean value and standard error of the means (SEM), and the result with *p* < 0.05 was considered statistically significant. When the effects of diet, breed, or diet × breed interactions were significant (*p* < 0.05), means were compared using the Duncan’s test.

## 3. Results

### 3.1. Nutrient Intake

The results of nutrient intake are summarized in Table 2. There were significant effects of diet (*p* < 0.001) on silage, OM, CP, starch, ADL, TP, CT, and metabolizable energy intake. The silage, OM, CP, and starch intake of the lambs fed with the SS diet was lower than the WS diet, but the ADL, TP, and CT intake was higher. The effect of sheep breed was also significant on alfalfa, concentrate, CP, and TP intake (*p* < 0.05). DP was found to have a higher alfalfa, concentrates, and CP intake, but the CT intake was lower than TH. There were significant interaction effects (*p* < 0.05) between diet and breed on TP and CT intake.

### 3.2. Growth Performance and Carcass Characteristics.

The results of growth performance and carcass characteristics are summarized in Table 3. The diet had a significant effect (*p* < 0.05) on final weight, ADFI, ADG, F/G, slaughter weight, HCW, and loin muscle area. Compared with the SS diet, WS diet significantly increased lamb’s ADFI, final weight, and ADG, and decreased its F/G (*p* < 0.001). The slaughter weight (*p* = 0.018) and HCW (*p* = 0.02) of the lambs fed with the SS was lower than that of the WS diet, but the loin muscle area was higher (*p* = 0.003) in the lambs fed the WS diet. The breed also had a significant effect (*p* < 0.001) on initial weight. The initial weight of the TH is significantly lower than that of the DP. There were no interaction effects of diet and breed on growth performance and carcass characteristics (*p* > 0.05).

### 3.3. Physical–Chemical Characteristics of Meat

The results of the physical–chemical characteristics of meat are summarized in Table 4. The diet had a significant effect on intramuscular fat (*p* = 0.018), ash (*p* = 0.080), pH_45 min_ (*p* = 0.002), and meat color (*p* < 0.01). The SS diet significantly increased the ash content and decreased the intramuscular fat content of lambs compared with the WS diet. The pH_45 min_ of the lambs fed with the SS diet was higher than lambs fed the WS, while the L*, a*, and b* values were lower in lambs receiving the SS diet. The sheep breed also had a significant effect on pH_45 min_ (*p* = 0.015), pH_24 h_ (*p* = 0.035), and meat color (*p* < 0.01). The pH_45 min_ of the TH group was higher than the DP, but the pH_24 h_ was lower. The L*, a*, and b* of the TH lambs were also higher than the DP. There were significant interaction effects (*p* < 0.05) observed between diet and breed for pH_45 min_ (*p* = 0.019), pH_24 h_, and b* (*p* < 0.001). The highest pH_45 min_ and pH_24 h_ was observed in the TH fed with SS diet, while the highest b* value was observed in this same breed fed the WS diet.

### 3.4. Meat Hydrolysate Amino Acids Profiles

The results of amino acid content (g/kg on dry matter basis) determined in LD are summarized in Table 5. Both diet and breed had no effect (*p* > 0.05) on the amino acid content of the meat. However, a higher content of essential amino acids (EAA) and total amino acids (TAA) was found in lambs fed the SS diet, 340.14 g/kg and 888.36 g/kg, respectively. In all groups of essential amino acids, lysine (57.72–71.38 g/kg) and leucine (52.54–65.05 g/kg) were the most represented, followed by arginine (39.94–47.89 g/kg). The ratio between essential/non-essential amino acids (E/N) in all groups was higher than 37%. The ratio between essential/total amino acids (E/T) was in the range of 60%–62%.

### 3.5. Intramuscular Fatty Acid Profiles

Table 6 reports the fatty acid composition (% of total fatty acid) of longissimus dorsi intramuscular lipids. The content of saturated fatty acid (SFA) was affected by both diet (*p* = 0.027) and breed (*p* = 0.043), and their interaction (*p* = 0.010). The highest SFA value was observed in meat from the TH fed the WS diet, while the lowest value was observed in this same breed fed with the SS diet. The content of C15:0 (*p* = 0.036), C16:0 (*p* = 0.039), and anteiso C17:0 (*p* = 0.043) was significantly affected by diet. And the higher C15:0, anteiso-C17:0 content was observed in meat from both the DP and TH fed with the SS diet while the C16:0 content was lower in lambs fed with the SS diet. Both diet and breed had no significant effect on total monounsaturated fatty acid (MUFA) (*p* > 0.05). Although, a significant interaction between diet and breed was observed (*p* < 0.001), and the lowest value was observed in meat from DP fed with the SS diet. The content of C18:1n9t was affected by diet (*p* < 0.001), and the highest value was found in the lambs fed with the SS diet. Diet also significantly affected the content of PUFA (*p* = 0.022), C18:2n6c (*p* < 0.001), and C18:3n-3 (*p =* 0.003) in intramuscular lipids, and the lambs fed with the SS diet had higher PUFA concentration than the WS diet. Furthermore, the C18:3n-6 was affected by the diet (*p* = 0.036), breed (*p* = 0.009) and their interaction (*p* < 0.001); the highest value was detected in TH fed with WS diet. There was a significant interaction between diet and breed on PUFA (*p* = 0.032) and C18:2n6c (*p* = 0.034), and the highest values were both found in meat from DP lambs fed the SS the diet.

## 4. Discussion

### 4.1. Growth Performance and Carcass Characteristics

Diet composition is the main factor associated with growth of ruminants in individuals of the same genotype [23]. Previous research has claimed that NDF and ADL concentration is negatively correlated with voluntary feed intake [24,25]. In the present experiment, the content of NDF and ADL in the SS diet was higher than in the WS diet, which may be the main reason for the low feed intake of the lambs fed the SS diet. Therefore, the lower feed intake of the lambs fed the SS diet could explain the difference in weight gain between the WS and SS groups. Additionally, Makkar reported that the CT may have negative effects on feed intake, protein digestibility, and animal performance [26]. However, the impact of CT on feed intake and growth performance has been controversial, as its effect depends on several factors including concentration and chemical composition, basal diet, animal species, and metabolic status [26]. Therefore, the CT contained in the SS diet may be another reason for the decrease in feed intake by lambs under the experimental conditions used in this study. The HCW of lambs has always been an important index to measure performance. However, in our study, the lambs fed the SS diet did not result in increased HCW, possibly due to the different ADFI of the SS and WS diets. Furthermore, the lower HCW of the lambs fed the SS diet may lead to a higher dressing percentage. Loin muscle area is highly positively correlated with lean meat rate, and always used for estimating the weight of lean meat [27]. Under our experimental conditions, the loin muscle area was not modified by the breed, but the diet’s effect was observed. This result is also consistent with the lower intramuscular fat content in lambs fed with SS diet.

### 4.2. Physical–Chemical Characteristics of Meat

Intramuscular fat has the most important role in the flavor and sensory characteristics of meat products [28]. In the present study, the DP and TH fed with the WS diet had the highest intramuscular fat content, which is more than 20%. This result was consistent with Ali Mujtaba Shah et al. [29]. It may be due to the starch intake of lambs fed the WS diet, which was higher than the SS diet. We speculate that more starch digestion may increase the level of glucose absorbed in the blood of ruminants, and increase the secretion of insulin (a hormone that promotes adipogenesis in all adipocyte tissues) [30,31], thus enhancing lipid accumulation in this group of lambs. Furthermore, Della Malva et al. reported that the pH values of the lamb meat are between the range of 5.5 to 5.8, which are considered normal levels for sheep meat 24 h after slaughter [32]. It should be emphasized that a normal pH suggests that other parameters indicative of the meat quality (such as color and tenderness) will have a good result because they will be affected by pH [33]. In the present experiment, all pH_24 h_ values conformed to the standard. Furthermore, the pH_45 min_ and pH_24 h_ of the TH fed with the WS diet was lowest. This effect can be explained by higher carcass weight of the lambs fed with the WS diet. Bonagurio et al. and Beriain et al. reported that higher muscle glycogen was reserved for heavy lambs (the weight between 25 kg and 45 kg), because being given ad libitum access to concentrates for a long period before slaughter encouraged rumen fermentation and the production of volatile fatty acids, particularly propionic acid, which is the precursor of muscle glycogen with a subsequent higher lactic acid production [33,34]. In this study, the higher starch intake in the WS group may lead to the accumulation of propionic acid in the rumen of ruminants [35]. However, the pH_45 min_ and pH_24 h_ of DP had no significant difference between the SS and WS diet, because of its final weight exceeding 45 kg. In addition, the authors found that lower carcass weight also increased meat’s shear force [36,37]. Purchas found that as the pH_45 min_ increased from 5.5 to 6.2, there was a decrease in sarcomere length, and shear force increased [38]. Interestingly, in the present study, we found that the shear force of TH lambs was higher than DP, but the carcass weight was lower, which may be due to the lower carcass weight’s increased effect on pH_45 min_, and thus shear force increased.

Meat color is the preferred apparent indicator for consumer judgments of meat quality [39], and dietary treatments also influence the colorimetric characteristics of meat [40]. Meat from the lambs fed the WS diet showed a higher L* value than meat from lambs fed the SS diet. This finding may be attributed to different intramuscular fat content between the groups, as confirmed in the literature [41]. In this study, meat from the TH fed the SS diet showed a lower L* value than the lambs fed the WS, and their intramuscular fat content was also found to be lower. In addition, Ledward et al. reported that meat pH_45 min_ values may also another reason that affects meat’s L* [42]. The a* intensifies as a consequence of the increasing concentration of myoglobin [43]. Meat from the lambs fed the SS diet showed a lower a* value than lambs fed the WS diet. This may be due to the different nutrient intake of lambs under different treatment conditions. The authors have observed that adding different tanniferous plant species in lamb diets initiates a reduction of hemoglobin in the blood [44]. The b* values were greater for the lambs fed with the WS diet, in agreement with those found in beef heifers by Casasús et al [45]. In this study, the colorimetric characteristics of meat were also influenced significantly by breeds, and the TH is lighter than DP lambs, which is in accordance with the results of Sañudo et al. and Santos-Silva et al., who observed the darkening of meat when slaughter weight increased [46,47].

### 4.3. Meat Hydrolysate Amino Acids Profiles

The composition of meat amino acids directly affected its nutritional values [48]. The meat protein content is a decisive factor in evaluating meat quality, especially its EAA content. In the present study, the E/T was about 38%, which met the human dietary requirements for amino acids listed in the World Health Organization’s report [49]. The EAA composition of the carcass can be used as a reference for the ideal protein (amino acid) requirements for lamb growth [50]. In the group of essential amino acids, lysine and leucine were the most represented. This experiment confirmed the larger quantity of the amino acids, which is consistent with the study by Miguélez et al [51]. Muscle proteins are composed of twenty-one amino acids, ten of which must be supplied by the diet. In this experiment, the CP intake of lambs fed the SS diet was lower than lambs fed the WS diet, but the amino acids were not significantly different in composition and ratio. This is inconsistent with the study by Wang et al., who reported that amino acids in LD muscle increase linearly with the increase in diet protein levels [52]. This may be related to the effect of CT on efficiency of ruminal protein metabolism [8]. Kamel et al. reported that as animal’s intake an appropriate level of tannins, the N release from the dietary protein degradation may be reduced in the rumen, thereby increasing the absorption of amino acids in the small intestine [53]. The amino acids in meat can also promote its aroma through the Maillard reaction between amino acids and carbonyl compounds during cooking [54]. For example, leucine and cysteine play an important role in aroma formation, because they provides Strecker aldehyde and aroma-potent thiophenes in the cooked meat system [54]. Therefore, the relative higher concentrations of amino acids, such as leucine, alanine, cysteine, and arginine, in meat from the SS group and the DP group could improve meat aroma. However, it is necessary to clarify the effect and potential mechanism in future research.

### 4.4. Intramuscular Fatty Acid Profiles

Diet significantly affects carcass characteristics, chemical composition, and also the fatty acid profile of muscles [55]. Under the conditions of this experiment, the diet was found to exert an influence on the total SFA content in all of the studied breeds, with total SFA content higher in TH fed with the WS diet, because of its higher content of C14:0, C16:0, and C18:0. Furthermore, the C14:0 and C16:0 of total SFA was lower in meat of the lambs fed the SS diet compared to WS diet. The effect of the diet on C14:0 and C16:0 content (both considered to be atherogenic fatty acids) was observed in all test breeds, indicating that no different responses to diet can be expected for each breed [56]. Regarding C18:0, it accounted for around 20% of the total fatty acids detected, and no significant differences between diets were observed for all breeds. This may be due to the intake of structural fiber content between the two diets, which were not significantly different [22]. Although SFA is generally considered unhealthy, some of them have positive effects, such as odd and branched chain fatty acids (OBCFA) which possess antitumoral activity and maintain the fluidity of cell membrane at low temperature [57,58]. However, OBCFA are generally found at extremely low levels in plant feeds, and they have a strong correlation with the content of volatile fatty acids in rumen of ruminants [59]. Vlaeminck et al. found that the ratio of acetate was positively correlated with iso C14:0, iso C15:0, anteiso-C15:0, and anteiso-C17:0 in milk [59]. In this experiment, the content of C15:0 and anteiso-C17:0 in the WS group were lower than that in the SS group. It may be that the high starch intake in the WS fed lambs leads to higher levels of propionate and lower levels of acetate in the rumen [34]. Total MUFA content accounted for about 45–46% of the total detected fatty acids, and the major MUFA for all groups was C18:1n-9. Regarding the MUFA content in the intramuscular fat of lambs, a diet and breed interaction was observed, showing that the response to dietary treatments is breed-related. According to Aldai et al., the effect of the breed could be different when individual fatty acid concentrations are compared [60]. In our study, feeding the WS diet could increase the MUFA content of DP, but it would not increase the MUFA content of TH. These results suggest that, with regards to the MUFA content in the intramuscular fat of lambs, the response to dietary treatment is also breed-related. In addition, the major MUFA in meat is C18: ln-9c, which is included in the calculations of total desirable fatty acids for human health [54,61]. It is widely believed that when animals are exposed to different diets, they undergo noticeable alterations on the 18:1 fatty acid metabolism [62,63]. However, in this experiment, the effect of the diets on C18: ln-9c of both breeds was not significant, but the content of C18: ln-9t of the SS group was higher than the WS group. Public health policies indicate that the intake of saturated FA (SFA) should be reduced and that the consumption of long chain PUFA fatty acids (PUFAs) should be increased in humans [64]. Abdelhamid et al. also concluded that increasing the intake of PUFA may slightly reduce the risk of coronary heart disease and cardiovascular disease events [65]. The absence of breed effects on the PUFA content in the current study is in agreement with a review by De Smet et al [66]. However, the PUFA was higher in meat of the SS group compared to the WS group. It may be due to sweet sorghum varieties having a moderate phenolic content [9]. The ability of dietary phenolics (e.g., tannins) to increase PUFA in ruminant meat has been widely reported [11,67]. Researchers pointed out that the addition of tannins could reduce ruminal biohydrogenation by inhibiting the proliferation of ruminal microorganisms, which could have produced higher escape of PUFA from rumen to tissue and meat. Furthermore, the interaction between diet and breed observed in PUFA shows that the influence of the diet on each breed can be different in terms of PUFA content. In addition, we did not detect CLA isomers and long-chain PUFA, which was consistent with the experimental results of other authors [68,69]. This may be due to the contents of CLA isomers and long-chain PUFA (e.g., arachidic acid, eicosapentaenoic acid, behenic acid) being too low to be detected in the animal LD muscle under the experimental conditions. Nevertheless, it is an extensively studied fatty acid for its positive implications for humans. Therefore, it is necessary to improve the identification and quantification methods of fatty acids in future research. In this experiment, the P/S ratio of the lambs fed the SS diet was higher than the lambs fed the WS diet due to a higher PUFA level of the SS diet. The P/S value of this study is lower than the recommended P/S ratio (0.4 to 0.5) [70]. However, this was not related to dietary treatments.

## 5. Conclusions

Under the conditions of the present study, most variables of growth performance and carcass traits were influenced more by diet than breed. Lambs fed the WS diet had better growth performance and fatter carcasses than those fed the SS diet. In terms of meat quality, the effect of diet on the meat quality of lambs seems to be breed-related. The results obtained in this study confirm that the influence of the diets on physical–chemical composition and variations in fatty acid profiles of longissimus dorsi is different between breeds (DP and TH). Lambs fed the SS diet had reduced intramuscular fat content, meat color, and concentration of SFA while improving the content of fatty acids, which is beneficial to human health.

## Figures and Tables

**Table 1:** Chemical composition of the experimental diets (g/kg on dry matter basis).

Item ^1^	Silage ^2^		Alfalfa Pellet ^3^	Concentrate ^4^
	Sweet Sorghum	Whole-Crop Corn		
OM	937.56	940.67	897.81	887.74
CP	37.30	41.70	84.00	154.00
NDF	672.53	556.99	620.61	191.43
ADF	416.08	339.95	424.79	83.94
Starch	18.00	81.30	20.10	150.00
ADL	32.20	19.00	79.90	9.60
TP	21.37	ND	NA	NA
CT	13.48	ND	NA	NA
Metabolizable energy (MJ/kg)	6.71	7.99	7.29	12.04

^1^ OM, organic matter; CP, crude protein; NDF, neutral detergent fiber; ADF, acid detergent fiber. ADL, acid detergent lignin; TP, total phenolics; CT, condensed tannins. ^2^ ND, not detected. ^3,4^ NA, not analyzed.

**Table 2:** Effect of silage diet and breed on nutrient intake of lambs (*n* = 7 per group).

Item ^1^	DP ^2^		TH ^3^		SEM ^4^	*p* Value		
	SS	WS	SS	WS		Diet	Breed	Diet × Breed
Feed intake (kg/day on dry matter basis)								
Silage	0.51 ^b^	0.61 ^a^	0.46 ^c^	0.58 ^a^	0.014	<0.001	0.447	0.338
Alfalfa	0.11 ^a^	0.11 ^a^	0.09 ^b^	0.09 ^b^	0.003	0.510	0.005	0.445
Concentrate	0.36 ^b^	0.38 ^a^	0.28 ^c^	0.29 ^c^	0.008	0.134	<0.001	0.171
Nutrient intake (g/day on dry matter basis)								
OM	897.20 ^b^	1011.45 ^a^	754.63 ^c^	886.60 ^b^	18.990	<0.001	0.526	0.253
CP	83.67 ^b^	92.92 ^a^	67.21 ^d^	76.70 ^c^	1.873	<0.001	0.046	0.408
NDF	481.20	483.12	415.29	435.21	7.256	0.401	0.419	0.215
ADF	289.91	250.82	287.64	260.08	4.300	0.736	0.418	0.208
Starch	65.24 ^c^	108.55 ^a^	51.76 ^d^	92.95 ^b^	4.332	<0.001	0.072	0.428
ADL	28.81 ^a^	24.21 ^b^	24.30 ^b^	20.93 ^c^	0.582	<0.001	0.388	0.105
TP	10.90	ND	14.40	ND	1.249	<0.001	<0.001	0.001
CT	6.83	ND	6.19	ND	0.632	<0.001	0.341	0.021
Metabolizable energy (MJ/day)	8.56	10.24 ^a^	7.06 ^d^	8.82 ^b^	0.226	<0.001	0.174	0.355

^a–d^ Means with different superscript letters in the same row differ significantly (*p* < 0.05). ^1^ OM, organic matter; CP, crude protein; NDF, neutral detergent fiber; ADF, acid detergent fiber; ADL, acid detergent lignin; TP, total phenolics; CT, condensed tannins. ^2^ DP, Dorper sheep; SS, Sweet sorghum silage; WS, Whole-crop silage. ^3^ TH, Thin-tailed Han sheep; ND, not detected. ^4^ SEM, standard error of means.

**Table 3:** Effect of silage diet and breed on growth performance and carcass traits of lambs (*n* = 7 per group).

Item ^1^	DP ^2^		TH ^3^		SEM ^4^	*p* Value		
	SS	WS	SS	WS		Diet	Breed	Diet × Breed
Growth performance								
Initial weight (kg)	33.20 ^a^	33.59 ^a^	25.96 ^b^	25.44 ^b^	0.797	0.648	<0.001	0.639
Final weight (kg)	45.31 ^a^	49.41 ^a^	34.76 ^c^	40.31 ^b^	1.301	<0.001	0.660	0.444
ADFI (kg/day DM)	0.98 ^b^	1.10 ^a^	0.82 ^c^	0.96 ^b^	0.021	<0.001	0.689	0.225
ADG (g/day)	113.69 ^b^	164.73^a^	91.64 ^b^	154.91 ^a^	8.499	<0.001	0.377	0.501
F/G	9.11 ^ab^	6.89^b^	10.00 ^a^	6.68 ^b^	0.496	0.003	0.105	0.365
Carcass traits								
Slaughter weight (kg)	44.39 ^ab^	47.79 ^a^	32.38 ^c^	38.08 ^b^	1.580	0.018	0.163	0.993
HCW (kg)	21.74 ^b^	24.48 ^a^	17.76 ^c^	19.29 ^c^	0.696	0.020	0.353	0.807
Dressing percentage (%)	59.81	57.58	58.69	55.92	0.705	0.262	0.064	0.849
Back fat thickness (mm)	5.04	4.93	5.31	3.33	0.378	0.133	0.858	0.120
Loin muscle area (cm^2^)	19.51 ^a^	15.69 ^ab^	15.97 ^ab^	14.04 ^b^	0.893	0.050	0.465	0.693

^a–c^ Means with different superscript letters in the same row differ significantly (*p* < 0.05). ^1^ ADFI, average daily feed intake; ADG, average daily gain; F/G, feed conversion ratio; HCW, hot carcass weight. ^2^ DP, Dorper sheep; SS, Sweet sorghum silage; WS, Whole-crop silage. ^3^ TH, Thin-tailed Han sheep. ^4^ SEM, standard error of means.

**Table 4:** Effect of silage diet and breed on physical–chemical composition of longissimus dorsi (*n* = 7 per group).

Item ^1^	DP ^2^		TH ^3^		SEM ^4^	*p* Value		
	SS	WS	SS	WS		Diet	Breed	Diet × Breed
Chemical composition (% on dry matter basis)								
Protein	71.90	71.33	74.16	72.85	1.613	0.801	0.612	0.920
IMF	19.99 ^ab^	23.33 ^a^	18.07 ^b^	20.83 ^ab^	0.696	0.018	0.070	0.800
Ash	4.36	3.61	4.24	4.10	0.129	0.080	0.450	0.223
Meat pH								
pH_45 min_	6.04 ^b^	5.91 ^b^	6.64 ^a^	5.92 ^b^	0.092	0.002	0.015	0.019
pH_24 h_	5.66 ^a^	5.75 ^a^	5.70 ^a^	5.47 ^b^	0.034	0.059	0.035	<0.001
Meat Color								
L*	51.20 ^b^	54.04 ^b^	53.96 ^b^	61.77 ^a^	1.288	0.003	0.002	0.241
a*	22.67 ^b^	24.46 ^b^	25.09 ^b^	32.15 ^a^	1.113	0.007	0.003	0.077
b*	11.28 ^b^	14.99 ^b^	12.30 ^b^	22.98 ^a^	1.751	<0.001	<0.001	<0.001
Drip loss (%)	1.25	1.44	1.33	1.68	0.132	0.202	0.538	0.703
Cooking loss (%)	36.43	34.78	36.46	36.66	0.502	0.495	0.372	0.386
Shear force (N)	2.50 ^ab^	2.05 ^b^	2.65 ^a^	2.65 ^a^	0.099	0.212	0.048	0.207

^a,b^ Means with different superscript letters in the same row differ significantly (*p* < 0.05). ^1^ IMF, intramuscular fat; L*, lightness; a*, redness; b*, yellowness. ^2^ DP, Dorper sheep; SS, Sweet sorghum silage; WS, Whole-crop silage. ^3^ TH, Thin-tailed Han sheep. ^4^ SEM, standard error of means.

**Table 5:** Effect of silage diet and breed on amino acid content (g/kg on dry matter basis) of longissimus dorsi (*n* = 7 per group).

Amino Acids ^1^	DP ^2^		TH ^3^		SEM ^4^	*p* Value		
	SS	WS	SS	WS		Diet	Breed	Diet × Breed
Total EAA	340.14	280.57	302.98	290.66	11.390	0.120	0.561	0.290
Isoleucine	39.46	31.76	34.52	34.59	1.313	0.119	0.614	0.169
Leucine	65.08	52.54	58.04	55.88	2.273	0.115	0.677	0.254
Lysine	71.38	57.72	63.41	60.90	2.495	0.115	0.624	0.264
Methionine	11.95	11.81	9.92	12.07	1.197	0.709	0.743	0.670
Phenylalanine	33.38	27.55	29.80	23.42	1.177	0.098	0.280	0.938
Threonine	36.10	29.35	32.50	31.63	1.270	0.149	0.793	0.257
Valine	42.72	34.98	37.99	37.51	1.349	0.132	0.674	0.180
Tryptophan	11.62	10.30	10.57	11.00	0.359	0.557	0.817	0.267
Histidine	28.43	23.55	26.13	24.17	1.153	0.169	0.725	0.544
Total NEAA	548.22	454.46	487.55	477.59	17.826	0.159	0.596	0.248
Arginine	47.89	39.96	43.33	42.33	1.621	0.191	0.740	0.304
Tyrosine	22.94	19.52	21.41	20.64	0.788	0.217	0.902	0.424
Alanine	52.05	40.55	41.47	40.14	2.197	0.135	0.195	0.229
Aspartic acid	78.80	64.02	69.64	69.44	2.601	0.155	0.712	0.166
Cystine	19.12	17.99	17.71	17.24	0.519	0.477	0.340	0.767
Glutamic acid	143.52	118.16	128.60	125.69	4.859	0.162	0.703	0.259
Glycine	33.64	30.28	31.70	31.17	1.043	0.398	0.817	0.537
Proline	121.22	99.46	106.46	104.66	4.132	0.165	0.560	0.234
Serine	29.04	24.51	27.22	26.29	1.001	0.208	0.990	0.400
TAA	888.36	734.03	790.52	768.26	29.061	0.149	0.533	0.245
E/T (%)	38.29	38.06	38.33	37.82	0.188	0.370	0.812	0.733
E/N (%)	62.06	61.44	62.18	60.88	0.482	0.374	0.834	0.747
DAA	367.84	304.78	324.67	320.83	11.743	0.164	0.559	0.213
FAA	256.49	210.66	229.97	223.90	8.738	0.153	0.703	0.265
BCAA	147.27	119.28	130.64	127.47	4.929	0.120	0.659	0.208

^1^ TAA, total amino acids; EAA, essential amino acid; NEAA, non-essential amino acid; E/T, ratio EAA/TAA; E/N, ratio EAA/NEAA; DAA, flavor amino acid; FAA, functional amino acid; BCAA, branched-chain amino acid. ^2^ DP, Dorper sheep; SS, Sweet sorghum silage; WS, Whole-crop silage. ^3^ TH, Thin-tailed Han sheep. ^4^ SEM, standard error of means.

**Table 6:** Effect of silage diet and breed on fatty acid composition (% total fatty acids) of longissimus dorsi (*n* = 7 per group).

Fatty Acids ^1^	DP ^2^		TH ^3^		SEM ^4^	*p* Value		
	SS	WS	SS	WS		Diet	Breed	Diet × Breed
Total SFA	51.10 ^b^	50.55 ^b^	50.28 ^b^	55.51 ^a^	0.740	0.027	0.043	0.010
C10:0	0.10	0.11	0.11	0.12	0.005	0.149	0.456	0.617
C12:0	0.10	0.10	0.07	0.07	0.008	0.823	0.107	0.940
C14:0	2.20 ^b^	2.50 ^a^	1.97 ^c^	2.37 ^b^	0.095	0.077	0.346	0.790
C15:0	0.28 ^a^	0.24 ^b^	0.26 ^ab^	0.22 ^b^	0.011	0.036	0.359	0.950
C16:0	25.11 ^b^	27.12 ^a^	25.91 ^b^	27.51 ^a^	0.427	0.039	0.460	0.797
anteiso-C17:0	2.19 ^a^	1.95 ^b^	2.07 ^a^	1.91 ^b^	0.048	0.043	0.398	0.658
C17:0	1.36	1.23	1.35	1.26	0.041	0.252	0.788	0.943
C18:0	20.62	19.06	19.56	21.20	0.396	0.954	0.478	0.051
Total MUFA	43.36 ^b^	46.71 ^a^	46.25 ^a^	41.88 ^b^	0.696	0.547	0.265	<0.001
C17:1	0.49	0.43	0.48	0.41	0.019	0.101	0.608	0.897
C18:1n9t	2.14 ^b^	1.55 ^c^	2.67 ^a^	1.17 ^c^	0.173	<0.001	0.655	0.022
C18:1n9c	41.47	43.75	41.58	41.55	0.563	0.341	0.373	0.328
Total PUFA	4.12 ^a^	2.35 ^c^	3.46 ^b^	2.60 ^c^	0.223	<0.001	0.284	0.032
C18:2n6c	3.67 ^a^	2.26 ^b^	2.88 ^ab^	2.41 ^b^	0.166	<0.001	0.127	0.034
C18:3n-6	0.08 ^a^	0.06 ^b^	0.07 ^a^	0.09 ^a^	0.006	0.036	0.009	<0.001
C18:3n-3	0.38 ^a^	0.22 ^b^	0.31 ^ab^	0.23 ^b^	0.022	0.003	0.388	0.186
P/S	0.08 ^a^	0.05 ^b^	0.06 ^ab^	0.05 ^b^	0.004	0.003	0.144	0.169
n-6/n-3	10.01	8.4	9.90	11.00	0.593	0.617	0.668	0.510

^a–c^ Means with different superscript letters in the same row differ significantly (*p* < 0.05). ^1^ SFA, saturated fatty acid; MUFA, monounsaturated; PUFA, polyunsaturated fatty acid; P/S, ratio PUFA/SFA; n-6/n-3, (C18:2n6c + C18:3n-6)/C18:3n-3. ^2^ DP, Dorper sheep; SS, Sweet sorghum silage; WS, Whole-crop silage. ^3^ TH, Thin-tailed Han sheep. ^4^ SEM, standard error of means.

## Data Availability

All data generated or analyzed during this study are included in this article.
